# Voluntary Rein Tension in Horses When Moving Unridden in a Dressage Frame Compared with Ridden Tests of the Same Horses—A Pilot Study

**DOI:** 10.3390/ani9060321

**Published:** 2019-06-06

**Authors:** Lara Piccolo, Kathrin Kienapfel

**Affiliations:** Department for Biology and Biotechnology, Ruhr University Bochum, 44801 Bochum, Germany; Lara.Piccolo@rub.de

**Keywords:** rein tension, riding, peak forces, welfare, dressage, horse

## Abstract

**Simple Summary:**

The aim of this pilot study was to evaluate the maximum rein tension that horses voluntarily adopt when wearing side reins set in dressage frame without a rider, and to compare that to rein tension in dressage frame with a rider. Without a rider, all horses maintained a rein tension force of approximately 1 kg in all gaits. For the same horses with a rider, rein tension force was significantly higher at approximately 3 kg on each side to maintain the dressage frame. Understanding and lowering the peak forces acting on the mouth of the horse could enhance equine welfare in daily riding practice.

**Abstract:**

Too much rein tension while riding may compromise the welfare of the horse. But who generates the tension on the reins—the horse or the rider? The primary aim of this pilot study was to evaluate the maximum rein tension that horses voluntarily maintain without a rider compared to rein tension with a rider. A secondary aim was to evaluate conflict behaviours in relation to rein tension. Thirteen horses were used, all fitted with customised “Animon” rein tension sensors (25 Hz, up to 600 N range), free-moving with side reins set in dressage competition frame with the noseline on the vertical. Rein tension was measured at the walk, trot, and canter in both directions in a round pen. The same horses were then ridden by their usual riders and completed the same task on a riding ground. Continuous video recordings were obtained to subsequently quantify the occurrence of conflict behaviours. The difference in mean maximum peak of rein tension with and without a rider for each gait was compared using the Wilcoxon Rank Sum test. Without a rider, rein tension was significantly lower (Wilcoxon T = 0, *p* < 0.01, 7.5 N ± 2.8 N) than with a rider (Wilcoxon T = 0, *p* < 0.01, 24.0 N ± 12.3 N). Regardless of the different rein tensions in the ridden exercise, all of the horses exhibited approximately the same amount of rein tension in the unridden exercise. The frequency of conflict behaviour was higher with a rider than without (11 ± 14 per minute vs. 2 ± 3 per minute; T = 4, *p* < 0.01).

## 1. Introduction

The influence of the rider on the horse’s mouth via the reins can represent an aversive stimulus, which can be used in negative reinforcement [1]. To determine the absolute values of rein tension as an aversive stimulus, rein tension measurements were performed. To achieve a finely tuned interspecific communication between horses and riders it is important to use the smallest possible intensity of rein tension. According to Eisersiö et al., the factors that are believed to influence rein tension are the gait, the rider’s position, the lesson ridden, the level of training of horse and rider, as well as the rider’s ability to follow the horse’s movements [2].

Very few published data (e.g., Egenvall et al.) are available to differentiate the rein tension that is contributed by the rider from that contributed by the horse [3].

Preuschoft performed rein tension measurements on dressage horses that had completed basic training (German A-L level). Rein tensions typically ranging from 20–60 Newtons (N), and at times peaks of up to 150 N, were reported. It was also found that tension varied regularly, depending on the horse’s head movement. The highest rein tension occurred at the trot, followed by the canter, with the lowest in the walk [4]. In addition, Preuschoft reported an oscillating pattern of rein tension corresponding with the natural nodding movement of the horse’s head, depending on the gait. This is a 4 cm to 5 cm vertical movement of the head, which is differently pronounced in walk, trot and canter [4]. Preuschoft and Gottstein observed that the oscillating pattern of rein tension in the different gaits occurred even in carriage horses without a rider [5].

Clayton et al. measured rein tension at the walk, trot and canter. Like Preuschoft, they found an oscillating pattern of rein tension depending on the gait. At the walk, which is a 4-beat gait, a peak occurred as each hoof contacted with the ground. At the trot, a 2-beat gait, the highest rein tensions occurred in the support phase of the diagonal leg pair (the hoofs of the diagonal leg pair contacting the ground), while the lowest tensions occurred during suspension phase of the trot (no hoof contacts with the ground). For the canter, a distinct peak occurred in the support phase of the diagonal pair of legs, which was surrounded by two smaller peaks [6].

König von Borstel et al. measured the voluntary acceptance of rein tension of various bitless bridles compared to a single-jointed snaffle bit [7]. The measurement was performed in a standing position by fixing the reins over the horse’s neck and stretching it downwards for a food reward, thus bringing tension to the reins. The differences in rein tension observed between the different bridles were not significant. In comparing breeds used in this work, the highest maximum voluntary rein tension occurred with ponies and cold-blooded horses (mean: 43.9 N), compared to warmblood and Thoroughbred horses (mean: 29 N and 28.6 N).

Christensen et al. examined the voluntary acceptance of rein tension by bridle-naïve young horses in a food enticement situation [8]. The rein tensions that the horses generated to reach highly palatable food were determined. It was found that the highest rein tensions occurred during the first test (mean value: 10. 2 N), while in the second and third tests rein tensions were lower (mean value test 2: 6.0 N; mean value test 3: 5.7 N). This suggests that the horses learned to avoid tension on the reins. With short reins, which leads to higher rein tensions, conflict behaviour (going against the reins, opening the mouth, lifting the head) was significantly more frequent.

Until now, the voluntary rein tension of horses in motion has not been specifically investigated. Based on existing results, however, it can be assumed that horses with high rein tension will show more frequent conflict behaviour.

In the present study, following the design used by Christensen et al. [8] and König von Borstel et al. [7], the primary aim was to determine the maximum rein tension that horses voluntarily maintained when moving in dressage frame without a rider. In addition, rein tension measurements were recorded with a rider in order to differentiate rein tension that may be contributed by the rider from that contributed by the horse. To evaluate the mental state of the horses, behaviours indicative of conflict (conflict behaviour) were recorded. König von Borstel et al. have indicated that the evaluation of conflict behaviour is a valid method to determine stress responses in horses [9]. It has been suggested that, in the exercising horse, conflict behaviour may be a more reliable indicator of stress than physiological measurements which are no doubt affected by the exercise [9]. Kienapfel et al. also used conflict behaviour in previous studies to evaluate discomfort in horses [10].

We assumed that horses exercised in dressage frame with side reins without a rider will self-maintain a relatively comfortable rein tension. We hypothesized that rein tension with a rider would be greater than that which is self-maintained without a rider. We also hypothesized that rein tension greater than that which was self-maintained would be associated with greater observable conflict behaviour. Further, we hypothesized that horses that self-maintain a relatively low rein tension when not ridden, would also show lower rein tension when ridden.

## 2. Materials and Methods

**Figure 1:**
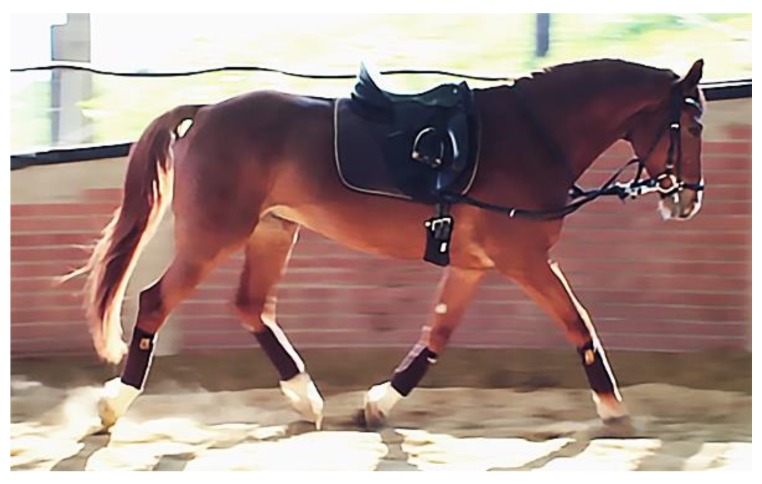
Head-neck-position during the unridden test.

**Figure 2:**
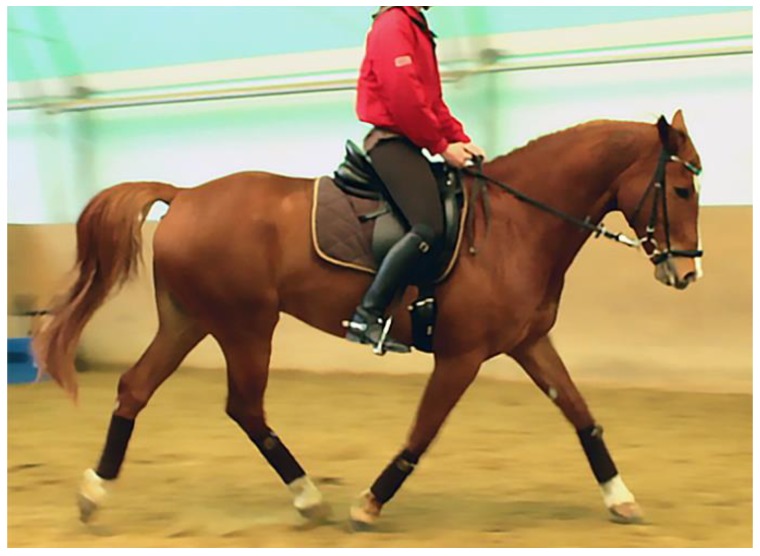
Head-neck-position during the ridden test.

Thirteen horses, all of whom had completed basic training (German A–L level) were used. Rein tension was measured using customized “Animon” rein tension sensors (25 Hz, up to 600 N range, via Bluetooth to a smartphone). For the unridden test, the horse was moving freely in a round pen (diameter of 18–24 m) with side reins fixed in competition frame with the noseline on the vertical (see Figure 1). Before the measurements started horses were individually warmed up by their usual rider. This included at least ten minutes of walking without side reins. This initial 10 min enabled the horse to habituate to the camera and to the two persons in the round pen (one person was the owner). After that, the side reins were installed so that the horse’s noseline was at the vertical or slightly in front of the vertical (up to 10°, means between 0° and 10°). The rein tension meters were attached between the side reins and the bit. Rein tensions were measured at the walk, trot, and canter in both clockwise and counter-clockwise directions. The direction was chosen in random order. The measurements started with walk, then trot, and then canter. For every gait at least 20 movement cycles in a steady head-neck position (confirmed from the video recordings) were measured. After that a similar set of readings was taken in the opposite direction. The horse’s movements were directed by their owner/handler. The same measurements were performed while ridden in their usual dressage frame by their usual riders on a circle with a diameter of 20 m in a riding ground (see Figure 2). The rein tension meters were installed between the bit and the reins. The measurements were started, stopped, and stored as an Excel-file with a smartphone app of “Animon.” All unridden exercises were done first and then the ridden exercise was second following immediately after tack change.

Video recordings of the exercises were obtained with a Canon EOS 600D (Canon Inc., Tokyo, Japan). The video recordings were started synchronously with the rein tension measurement. The video was subsequently reviewed to record each occurrence of conflict behaviour. The mean recording time for the unridden test for each direction was 63.35 s ± 16.81 s and for the ridden test for each direction was 79.96 s ± 24.00 s. The recording time varied because the head-neck positions of some horses were not steady. In some instances, additional seconds were recorded to ensure at least 20 movement cycles per horse and gait with a steady head-neck position with the noseline at the vertical. Each maximum peak of rein tension, which was observed as a step in walk, trot or canter in the video recording, was extracted using a self-written script (MATLAB and Statistics Toolbox Release 2012b, The MathWorks, Inc., Natick, MA, USA). The mean and standard deviation of the maximum peaks were calculated for each gait, direction, test, and horse. The rein tension data were not normally distributed (Shapiro-Wilk test: W = 0.92, *p* = 0.23). Accordingly, differences between the means of the maximum peaks of the rein tension for the ridden and unridden test, as well as between the different gaits and the directions, were evaluated using the non-parametric Wilcoxon Rank Sum test. In addition, the association of rein tensions during the ridden and unridden tests was evaluated using the Pearson correlation test.

Conflict behaviours, as defined in Table 1, were recorded for the duration of the exercises, using the focal animal method and translated into an ethogram for each horse [11]. Frequency of conflict behaviours per minute was calculated. Differences between the ridden and unridden tests were evaluated using a one-tailed Wilcoxon Rank Sum test.

Ethics: this study design was approved by the welfare officer of the Ruhr University Bochum (Tierschutzbeauftragter). According to German law this work is not considered an animal experiment and therefore needs no further approval. The horses were handled without causing pain or discomfort defined by law and used in their normal every day routine.

## 3. Results

### 3.1. Results of Rein Tension Measurements

**Figure 3:**
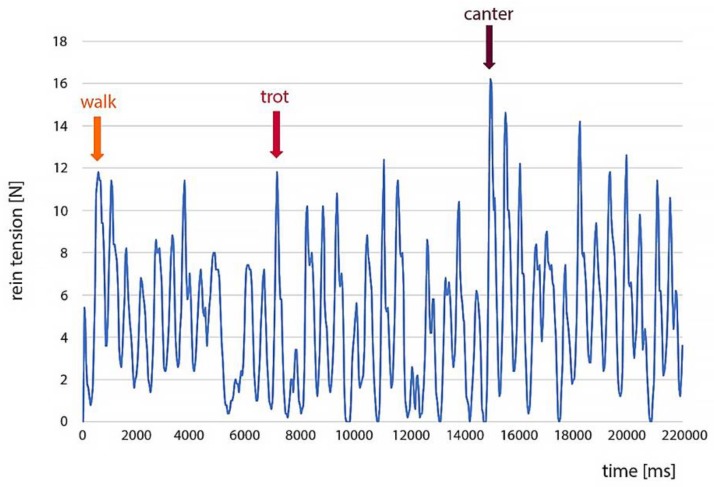
Raw data of a rein tension measurement while riding on the left hand; the data show an oscillating pattern for each gait; the first arrow indicates the start of the walk-phase, the second arrow indicates the transition from walk to trot, and the third arrow indicates the transition from trot to canter.

Figure 3 represents an example of raw tension data for the right rein during a measurement while riding on the left rein. Comparing these data to the video, each peak in rein tension corresponds to a step in walk, trot or canter. The first arrow indicates the beginning of the walk. During the walk, each peak in the rein tension corresponds to a footfall. The rein tensions then dropped to a minimum (trough in the trace) and then formed a peak with the subsequent footfall. The second arrow indicates the transition from walk to trot. Two peaks could be observed per movement cycle. The third arrow indicates the transition from trot to canter. Each canter cycle had a distinct peak, which was surrounded by two smaller peaks.

**Figure 4:**
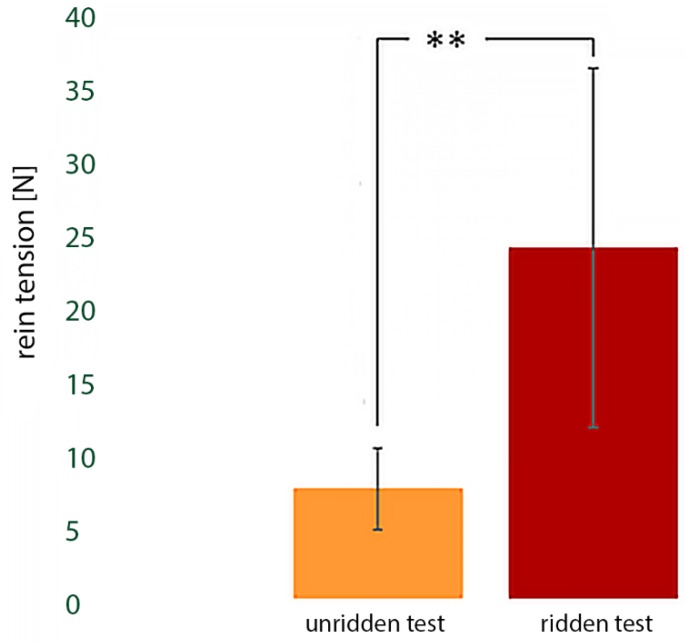
Mean values of the maximum peaks of rein tension in the unridden and ridden tests; the rein tension in the ridden test (24.0 N ± 12.3 N) is significantly (*p* < 0.01) higher than the rein tension in the unridden test (7.5 N ± 2.8 N); ** indicates *p* < 0.01.

Maximum rein tensions when unridden were significantly (7.5 N ± 2.8 N) lower than when ridden (24.0 N ± 12.3 N, T = 0, *p* < 0.01, see Figure 4). Differences in rein tensions between the two directions were not significant, neither in the unridden test (counter clockwise: 7.2 N ± 2.3 N, clockwise: 8.0 N ± 3.4 N) nor ridden test (counter clockwise: 22.0 N ± 9.6 N, clockwise: 25.1 N ± 14.6 N). Similarly, the difference between the right and left rein without rider (left rein: 7.2 N ± 3.2 N, right rein: 8.0 N ± 2.5 N) or with the rider (left rein: 23.4 N ± 12.9 N, right rein: 24.6 N ± 12.2 N) were not significant.

**Figure 5:**
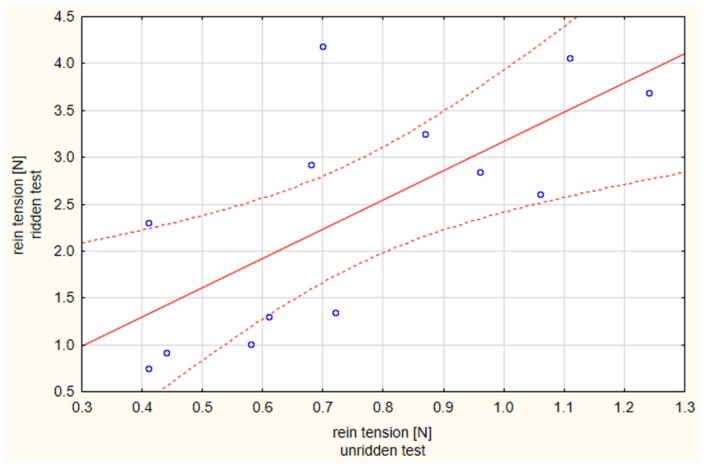
Correlation between the rein tension in the unridden and ridden state; correlation coefficient r = 0.702, *p* < 0.05; a positive linear relation between the rein tensions in the unridden and ridden state is shown in the scatterplot, circles represent results of individual horses; the dotted lines indicate the 95% confidence interval.

**Figure 6:**
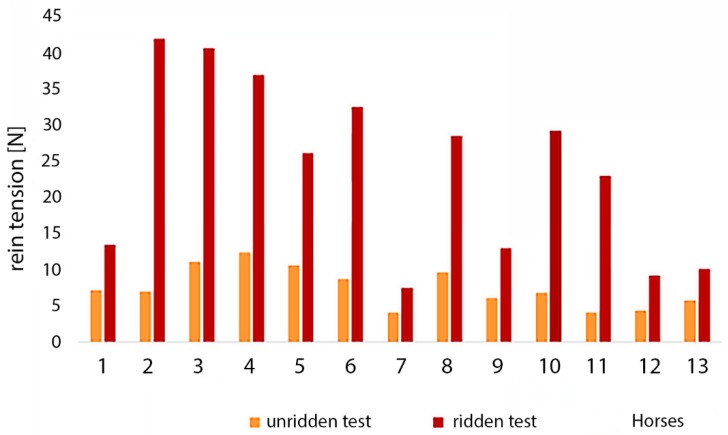
Rein tensions of each of 13 horses for each state; for each horse, except of Horse 7, the rein tension in the unridden test was significantly (*p* < 0.05) lower than in the ridden exercise.

As illustrated in Figure 5, rein tensions in the unridden and ridden tests were positively correlated (Pearson: 0.702). In addition, as illustrated in Figure 6, the mean values of the maximum peaks of the unridden and ridden exercise from each horse were compared. For all horses, except Horse 7, there was a significant difference between the lower rein tension when not ridden and higher tension when ridden (*p* < 0.05).

**Figure 7:**
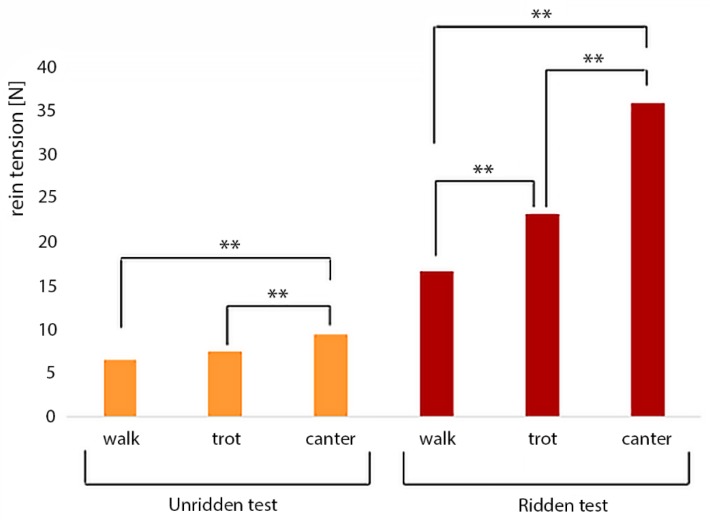
Mean values of the maximum rein tension of all horses in the different gaits; with a rider, the highest rein tension occurred during the canter (35.9 N ± 18.1 N) followed by the trot (23.2 N ± 14.1 N) and the walk (16.5 N ± 10.2 N); without a rider, rein tension was significantly higher during the canter (9.4 N ± 3.0 N) than the trot (7.4 N ± 3.2 N) as well as the canter and the walk (6.5 N ± 2.8 N), but not between walk and trot; ** indicates *p* < 0.01.

Figure 7 illustrates the rein tensions during the different gaits. For the unridden test, the mean maximum rein tension during canter (9.4 N ± 3.0 N) was significantly (*p* < 0.01) higher than during walk (6.5 N ± 2.8 N) or trot (7.4 N ± 3.2 N). For the ridden test, the mean rein tension differed significantly among the three gaits (*p* < 0.01). The lowest rein tension occurred during walk (16.5 N ± 10.2 N), followed by the trot (23.2 N ± 14.1 N), with the highest maximum rein tension during the canter (35.9 N ± 18.1 N).

### 3.2. Analysis of the Conflict Behaviour

**Figure 8:**
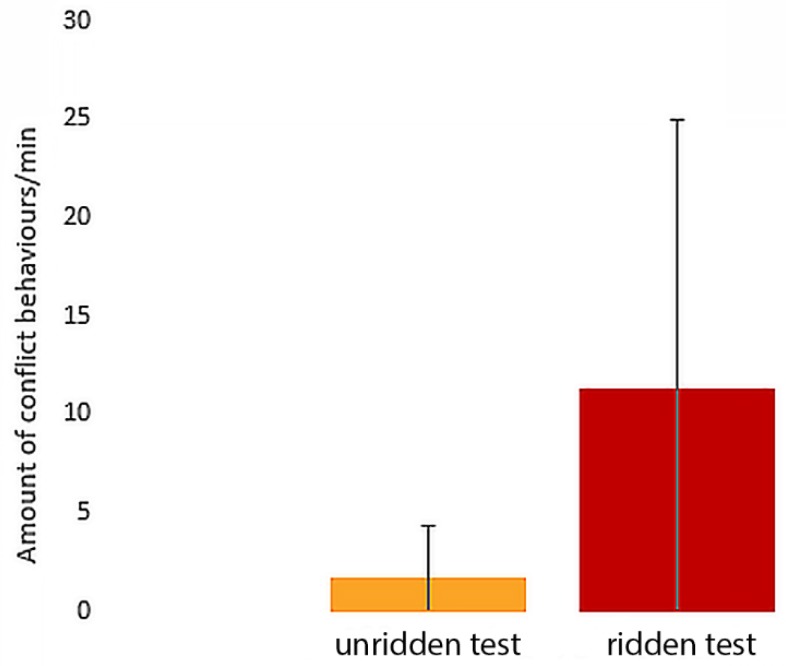
Mean number of observed conflict behaviours per minute in the unridden (2 ± 3) and ridden exercises (11 ± 14).

Without a rider, seven out of the 13 horses showed conflict behaviours compared to 11 of 13 when with a rider. As illustrated in Figure 8, the mean frequency of conflict behaviours per minute was more than five times higher with a rider (11 ± 14) than without (2 ± 3). The difference is significant (T = 4, *p* < 0.01).

**Figure 9:**
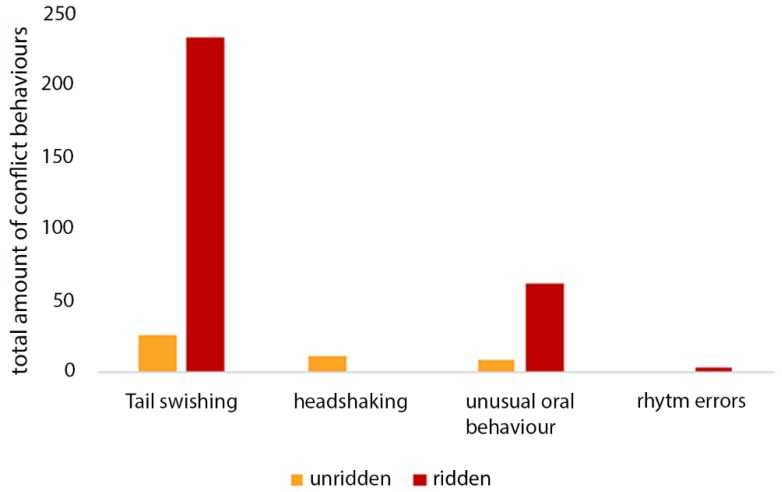
Total frequency of conflict behaviour of all horses shown per minute for each state and direction; headshaking occurred only during the unridden state whereas rhythm errors occurred only during the ridden exercise.

The specific conflict behaviours observed included headshaking, tail swishing, unusual oral behaviour and rhythm errors. Bucking, rearing, ears fixed backwards and going against the reins were not observed in either exercise. Figure 9 illustrates the total frequency of each conflict behaviour observed during each test. Tail swishing and unusual oral behaviour were more frequent during the ridden exercise (234 instances of tail swishing: 234, 62 instances of unusual oral behaviour) compared to the unridden test (26 instances of tail swishing, eight instances of unusual oral behaviour). Headshaking occurred only in the unridden test (11 instances). Rhythm errors were observed only during the ridden test (three instances).

## 4. Discussion

In this study the rein tension was compared during unridden and ridden tests. Four hypotheses were tested. The first hypothesis was that horses which move without a rider will adopt the most comfortable rein tension. The voluntary rein tension exhibited by these horses when unridden was 7.5 N ± 2.8 N. This is similar to the voluntary rein tension in young, inexperienced horses standing searching for a reward against restraining reins, with a mean maximum force of 10.2 N [8]. This may suggest that the mouths of the horses react equally sensitively to rein tension regardless of whether standing or moving.

These results also suggest that it is possible to maintain the correct dressage position with the nasal line on or slightly in front of the vertical with relatively low rein tension, i.e., below 10 N. Since rein tension may be an aversive stimulus that can cause discomfort or pain, it is important to keep the strength of this stimulus as low as possible. It might therefore be assumed as ideal that while riding, the horse’s self-selected rein tension when equipped with side reins should not be exceeded.

The second hypothesis was that horses ridden with greater rein tension than maintained during the unridden situation would show increased conflict behaviours. The number of conflict behaviours per minute with a rider was about five times greater than without a rider, which provides evidence in support of this hypothesis. Higher numbers of conflict behaviours can indicate discomfort and/or stress, so being ridden could be perceived as stressful by the horses in this study. Since the head-neck position remained the same for both states, with the noseline at or slightly in front of the vertical, and only the rider was added, it is reasonable to assume that factors related to the rider are the key difference. Higher rein forces while riding were measured in 12 of 13 horse-rider pairs; and the average rein tension was significantly (*p* < 0.01) higher when ridden than the self-selected rein tension during moving freely. It is reasonable to assume that rein tension greater than that which was adopted without a rider may be potentially painful. Of course, there are other aspects of carrying and interacting with a rider that could contribute to discomfort and/or stress. Nonetheless, an improvement of the level of rein tension while riding could lead to less discomfort and therefore fewer expressions of discomfort. The ability of horses to learn depends, among other things, on their mental state [12,13]. It has been reported that anxiety, for example caused by high pressure on tongue and jaw, results in occurrence of “learning blocks” [12,14]. Therefore, a lighter, more moderate use of the reins could also positively influence learning. A stimulus via the reins should be given with the lowest possible amount of tension, followed by an immediate release after the desired reaction has been achieved [1].

The third hypothesis was that the horses that maintained low rein tensions without a rider should react sensitively to greater rein tension with a rider; additionally, horses with high tension levels without a rider should exhibit higher rein tensions with a rider. The correlation was indeed significant. The lower the rein tension during the unridden test, the lower the rein tension was during the ridden test. Even while the mean maximum forces of the unridden horses were in a region below 10 N (very low if compared with the results of Preuschoft [4]) a prognosis can be made for the responsiveness to the reins under the saddle. This is a further indication, that the horses that are particularly sensitive in their mouths, and also the horses perceived as “hard mouthed” by their riders, can be ridden with relatively low rein tension, if introduced properly. There were individual differences among horses, but only in the ridden exercise did they lead to clearly higher rein tension in contrast to the unridden exercise. These results again point out the importance of the rider knowing about learning theory and how to correctly introduce a rein signal to the horse. Seeing only minor differences between the horses in the unridden situation may tempt riders to ignore individual differences, but on the contrary they provide a great opportunity for horse owners to learn about the character and learning type of their horse. As a pilot study, the number of horses in this study was small. With greater numbers, a database could be developed for riders to classify their horse as to “mouth sensitivity type” by means of their rein tensions when unridden. This could help to give personality-specific rein stimuli and monitor the development of mouth sensitivity of each horse in time, hopefully to a “lighter” category.

Preuschoft showed that rein tension oscillates in a regular way in each motion cycle and in a certain pattern depending on the gait [4], as did Clayton [6]. At the walk, there was one peak of the rein force in each step, i.e., when a front leg was touching the ground. At the trot, two peaks per movement sequence, and in canter one peak surrounded by two smaller peaks were found [6]. These results were also observed in the present study. Based on a video analysis it could not be conclusively clarified whether the peaks of the rein forces occur in the suspension phase as indicated in one study [15] or during the support phase of the diagonal leg pair of the trot as indicated in other studies [4,5,6].

Being a pilot study, a limitation is the small number of horses. Due to the described difficulties with the failure of showing heart rate variability because of problems with data acquisition and specific data analysis, the study sample was smaller than originally planned. Another heart rate monitor will be considered for a future study. Another influence could be that the ridden and unridden exercises were done in different locations, with unridden in a round pen and the ridden in an open riding arena. Although being used to all locations, this difference could have influenced the results and should be changed for further studies. However, the results were congruent within the dataset and in general with the literature. Since the feasibility is shown, the study will be expanded with higher numbers and small refinements in the study design as discussed to provide a broader insight into horse personality. A further goal is to make the next step into building a large database on finding the acceptable rein tension for most horses, which does not adversely affect their welfare.

In conclusion, horses exercised in a dressage frame adopted a lower rein tension without a rider than with a rider. Higher rein tension was associated with a higher frequency of behaviours indicating discomfort and/or stress. The results indicate that the higher rein tension may be primarily generated by the rider. The results suggest the potential to determine the amount of rein force exhibited by the horse and the rider, which provides interesting insights into the horse-rider interaction and may be a useful tool for the individual training of the rider.

## Figures and Tables

**Table 1:** Ethogram of conflict behaviour, based on sources: [10,12].

Behaviour	Process
Rearing	The horse’s forebody and forelimbs rise, such that the weight is borne on the hind limbs
Bucking	The horse lowers the head and neck and raises the hindlimbs off the ground
Unusual oral behaviour	The horse opens his mouth so a gap between the upper and lower jaw is visible, showing his teeth or tongue for more than 1 s
Tail swishing	The horse moves the tail in a fast motion in a vertical or horizontal direction or combined, e.g., after a signal given by the rider
Ears fixed backwards	The ears set back towards the neck
Headshaking	The horse moves the head quickly up-and downwards and/or from side-to-side
Going against the reins	The horse pushes the heads towards the ground or upwards resulting in extending the length of the reins
Errors in rhythm	Insertion of an additional step resulting in loss of the rhythm
